# Long-term development of lens fluorescence in a twin cohort: Heritability and effects of age and lifestyle

**DOI:** 10.1371/journal.pone.0268458

**Published:** 2022-05-26

**Authors:** Jakob Bjerager, Sami Dabbah, Mohamed Belmouhand, Line Kessel, Jesper Leth Hougaard, Simon P. Rothenbuehler, Birgit Sander, Michael Larsen

**Affiliations:** 1 Department of Ophthalmology, Rigshospitalet, Glostrup, Denmark; 2 Department of Ophthalmology, Odense University Hospital, Odense, Denmark; 3 Faculty of Health and Medical Sciences, Department of Clinical Medicine, University of Copenhagen, Copenhagen, Denmark; 4 Department of Clinical Sciences, Ophthalmology in Malmö, Lund University, Malmö, Sweden; 5 University Hospital Basel, Basel, Switzerland; Girne American University - Karmi Campus: Girne Amerikan Universitesi, CYPRUS

## Abstract

The blue-green autofluorescence of the ocular lens increases with age, glycemia and smoking, as the irreplaceable structural proteins of the lens slowly accumulate damage from the encounter with reactive molecular species. We have conducted a prospective study of lens autofluorescence over two decades in a twin cohort. The study included 131 phakic, non-diabetic adult twins (median age at follow-up 58 years, range 41–66 years) who were examined twice at an interval of 21 years. Change in anterior lens peak autofluorescence was analyzed in relation to age, current and baseline glycemia, cumulative smoking and heritability. The level of lens autofluorescence in the study population increased as a function of age and smoking (p ≤.002), but not as a function of glycemia (p ≥.069). Lens autofluorescence remained a highly heritable trait (90.6% at baseline and 93.3% at follow-up), but whereas the combined effect of age and cumulative smoking explained 57.2% of the variance in lens autofluorescence at baseline in mid-life, it only accounted for 31.6% at follow-up 21 years later. From mid to late adulthood, the level of blue-green fluorescence remained overwhelmingly heritable, but became less predictable from age, smoking habits and glycemic status. Presumably, as the lens ages, its intrinsic characteristics come to dominate over environmental and systemic factors, perhaps in a prelude to the development of cataract.

## Introduction

Ageing of the human lens is associated with continuous processes of change, including yellowing, decreasing blue light transmission, stiffening and enlargement, with concomitant changes in the form of presbyopia and gradually decreasing optical quality. This description covers diffuse changes, not the structural irregularities that are classified as cataract. Yellowing, and its extension browning, is accompanied by autofluorescence, which appears to result from spontaneous post-translational modifications of long-lived structural lens proteins by oxidation, aggregation of advanced non-enzymatic glycation end-products (AGEs) and the formation of high-molecular-weight aggregates [1–4]. The rate of glycation is proportional to glucose concentration and is therefore accelerated by hyperglycemia [2,5]. Environmental factors that contribute to post-translational protein modification include smoking, dietary intake of burnt or roasted foods and exposure to ultraviolet light [6–10].

Lens fluorescence can be measured quantitatively and has been done mainly at the blue/green excitation and emission wavelengths used to detect fluorescein. In adults, lens fluorophores in this spectrum seem to be dominated by AGEs, perhaps most prominently argpyrimidine and pentosidine, which accumulate with age [10–12]. Because the lens is optically transparent and there is little or no turn-over of a very large fraction of the proteins in the lens, measurement of lens fluorescence offers an opportunity to study a subject’ life-long exposure to protein-degrading factors, including metabolic and photooxidative stress and glycemia [13,14]. This has been of particular interest in diabetes mellitus [15], although clinical utility has been limited due to a considerable interindividual variation in lens fluorescence that can only partially be explained by known covariates [14]. A study of healthy subjects did not find evidence of a significant association of lens fluorescence with accommodative amplitude [16–18], but other studies have shown an association with nuclear cataract [19–23]. Because lens fluorophores tend to be yellow, it has been suggested that lens fluorometry may be useful for the assessment of cataract development [20,21]. Other than being of interest for the optical and mechanical properties of the lens, lens fluorescence is also a confounding element that has to be minimized in fundus fluorescence and autofluorescence imaging [1,24–27].

We have conducted a prospective 21-year follow-up twin study of lens fluorescence in healthy adult twins to assess the development of lens fluorescence in relation to age, glycemia, smoking and heritability, with the aim of quantifying contributions to interindividual variance in fluorescence.

## Materials and methods

### Study population

Subjects recruited from the Danish Twin Registry, who were examined at a baseline study in 1998 at the Department of Ophthalmology, Herlev Hospital, Denmark (n = 228), were invited to participate in its sequel, the 2019 Copenhagen Twin Cohort Eye Study. The participants of the baseline study were recruited from the GEMINAKAR study population (Danish Twin Research Center, University of Southern Denmark).

### Inclusion and exclusion criteria

The GEMINAKAR study excluded individuals with known diabetes or cardiovascular disease, conditions making a progressive maximal bicycle test impossible, pregnancy, or breast feeding. If one twin in a pair did not respond or was not willing to participate in the GEMINKAR study, the pair as such was excluded [28]. For the baseline lens fluorescence study, specific extra exclusion criteria counted cataract or other opacities of the refractive media within 2 mm of the optical axis of the eye [14]. Exclusion criteria for participation in the follow-up study were 1) ocular pathology or opacities that impaired imaging of the eye, 2) uncontrolled glaucoma with IOP >30 mmHg despite relevant treatment or 3) cognitive impairment that prohibited understanding and signing the informed consent form. Excluded from analyses were pseudophakic eyes, subjects in which less than 3 successful lens autofluorescence measurements could be obtained, and subjects with diabetes defined as HbA_1c_ ≥ 48 mmol/mol at the time of examination or a history of type 1 or 2 diabetes. Subjects with diabetes were excluded because it was assumed that their glycemia history could not be reliably charted and modelled.

### Data sources

Lens fluorescence at follow-up was measured in the right eye of phakic subjects using a commercial ocular fluorometer (Fluortron Master TM-2 with Windows software, revision B.17, OcuMetrics, Mountain View, California, USA), approximately 1 hour after dilation with tropicamide 1% eye drops. Excitation wavelength was 430–490 nm and detection was at 530–630 nm. Results are reported as ng/ml equivalent fluorescein concentration units in water at physiological acidity. The device was fitted with an anterior segment adaptor which allows the measurement of blue-green fluorescence at 149 incremental steps of each 0.125 mm along the optical axis of the anterior part of the eye. Measurements were performed in a dark room. Absorption-corrected anterior lens peak fluorescence was calculated using the manufacturer’s software, in which calculation algorithms are based on the principles of lens fluorescence peak extrapolation first described by Zeimer et al. [29]. Subjects were scanned up to six times in the attempt to achieve three successful scans. Unsuccessful scans counted scans with ambient background light values above 30% of the posterior absorption-corrected lens peak fluorescence, as recommended by the manufacturer, or if blinking had occurred at critical points during the scan.

Blood samples obtained at the follow-up visit were analyzed for HbA_1c_ (mmol/mol), whereas glycemia data at baseline was in the form of 2 hour oral glucose tolerance test (OGTT) concentrations (mmol/mL).

Data on smoking habits (smoking status as “yes”/”no”/”previous” and smoking pack years) were obtained by interviewing study participants. A smoking pack year was defined as 20 cigarettes smoked per day for one year. Individuals with <1 pack years were considered non-smokers.

Cross-sectional data from the baseline examination were acquired from data-archives at the Department of Ophthalmology, Rigshospitalet Glostrup, Denmark and data decryption keys were provided by the Danish Twin Registry, University of Southern Denmark, Odense, Denmark.

### Outcome measures and covariates

Lens fluorescence was tested for twin pair interrelatedness and relations to age, current glycemia (Hba_1c_ at follow-up and OGTT at baseline) and smoking pack years. Outcomes were heritability of lens fluorescence and correlations between study parameters.

### Statistical analysis

Statistical analyses were performed in R-Studio v1.2.5001 for Windows. Normality was tested by Shapiro-Wilk normality test. Parametric data were reported in means with 95% confidence intervals or standard deviations (SD) and compared using Student’s t-test. Non-parametric variables were reported in medians and interquartile ranges (IQR) and/or full ranges and compared using the Mann-Whitney U test. Lens fluorescence was transformed by log10 to obtain normal distributions in all analyses. All lens fluorescence values reported have been back-transformed by antilog to geometric means with 95% confidence intervals.

Univariate and multivariable log-level linear mixed model analyses adjusted for twin-pair data clustering were performed with the R functions ‘lmer’ (lme4 v.1.1.26 package) and ‘modelTest’ (JWileymisc v. 1.2.0 package) with adjusted R^2^ values reported, and coefficient estimates exponentiated and converted to percentages to designate the percentage increase in median lens fluorescence per one-unit increase in each covariate. Only covariates with statistically significant influence on lens fluorescence as found by univariate analysis were included in the corresponding multivariable analyses.

Broad-sense heritability analyses of lens fluorescence were performed with the R function ‘twinlm’ (mets v. 1.2.8.1 package) with adjustment for covariates that were found to have statistically significant effects on lens fluorescence in univariate linear regression analyses. Heritability and environmental influences were quantified into the coefficients A (additive genetics), D (dominant genetics), C (shared environment) and E (non-shared environment) and the broad-sense heritability coefficient *h*^*2*^ (A+D), calculated in each of the following combinatory heritability models: ACE, ADE, AE, DE and CE. Best-fitting heritability models were found by Akaike’s information criterion (AIC), with the lowest AIC-value defining the best-fitting model in each analysis group. Models with AIC-values between the value of the best fitting model and the value of the best fitting model plus two AIC-units were considered statistically non-inferior to the best fitting model. P-values below 5% were considered statistically significant in all analyses.

### Ethics

Study participants provided written informed consent. The study was approved by the regional Health Research Ethics Committee (No. H-18052822), Danish Data Protection Agency (No. VD-2018-434) and complied with the tenets of the Declaration of Helsinki.

## Results

**Fig 1:**
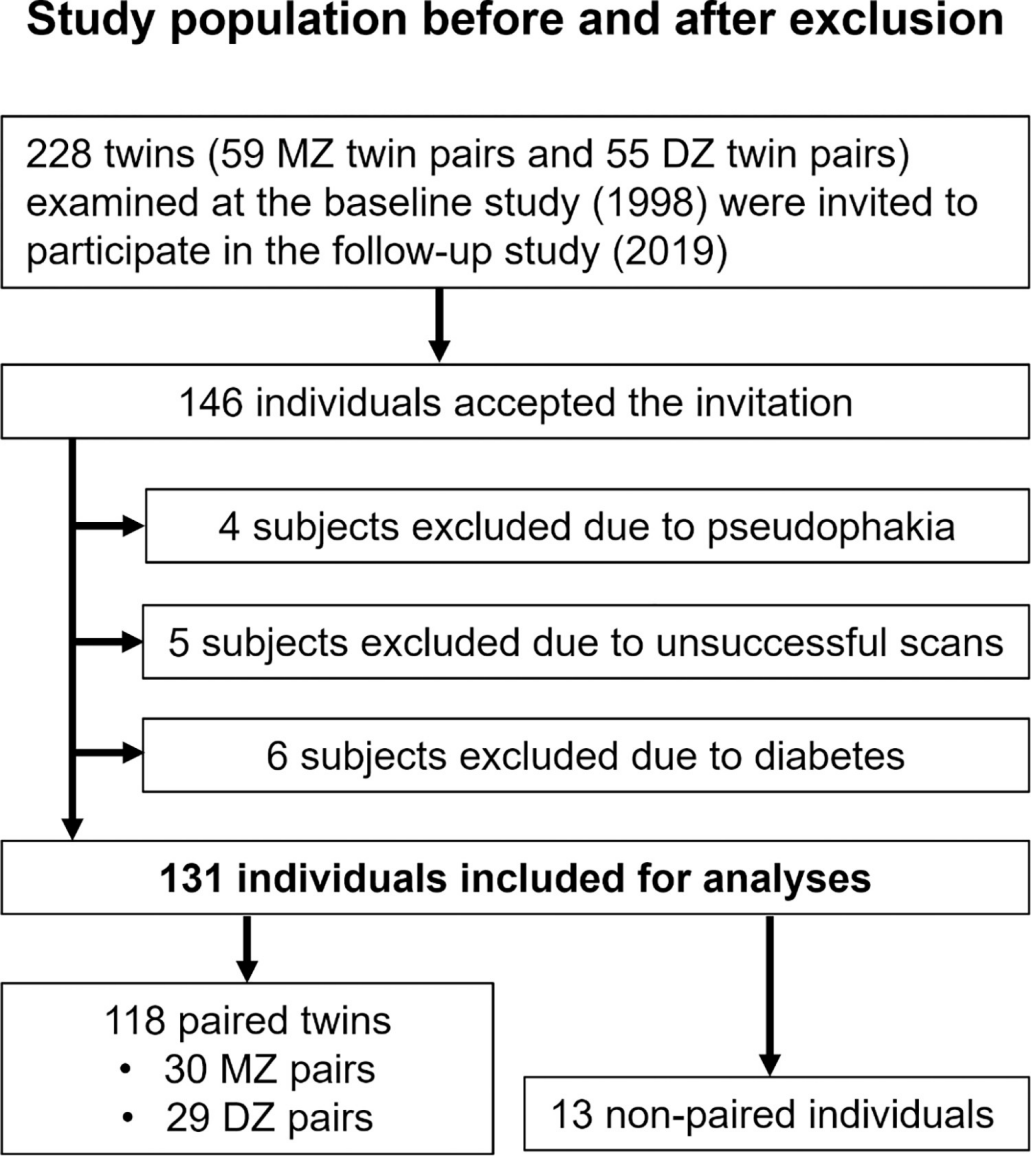
Study population before and after exclusion.

The invitation to participate in the follow-up study was accepted by 146 individuals of which 131 were included for analyses (Fig 1). The final study population consisted of 60 paired monozygotic twins (30 pairs), 58 paired dizygotic twins (29 pairs) and 13 non-paired study participants (participants whose twin was lost to follow-up or excluded due to our exclusion criteria).

The median age of subjects (n = 131) was 37 years at baseline (IQR 32–42, range 20–46) and 58 years at follow-up (IQR 53–63, range 41–66). Lens fluorescence had increased by a mean of 11.8 ng/ml pr. year [95% CI 10.7–12.9] from 293 ng/ml [273–314] at baseline to 529 [497–562] ng/ml at follow-up, which was a statistically significant increase (p < .001). Cumulative smoking had increased, but not significantly (p = .27). All study parameters were comparable for monozygotic and dizygotic twins, both at baseline (lens fluorescence, age, 120 min OGTT glycemia, smoking pack years) and at follow-up (lens fluorescence, age, HbA_1c_, smoking pack years) (all p > .11, Table 1).

**Table 1:** Characteristics of study population.

Parameter \ Population	Total population (MZ, DZ and non-paired)	P-value (baseline vs follow-up)	MZ population	DZ population	P-value (MZ vs DZ)
N	131	-	60 (30 pairs)	58 (29 pairs)	-
Sex, females, %	55.7%	-	53.3%	51.7%	-
**Baseline visit (1998)**					
Lens fluorescence, ng/ml, mean (95% CI)	293 (273–314)	-	293 (265–324)	294 (263–327)	.98
Age, years, median (IQR)	37 (32–42)	-	36 (31–41)	38 (34–42)	.26
120 min OGTT, mmol/L, mean (SD)	5.9 (± 1.1)	-	5.9 (± 1.1)	5.9 (± 1.2)	.86
Smokers (current or previous), %	36.6%	-	41.7%	32.8%	-
Smoking pack years if smoker, median (IQR)	6.6 (3.0–10.4)	-	7.0 (4.9–10.5)	6.4 (2.0–9.9)	.32
**Follow-up visit (2019)**					
Lens fluorescence, ng/ml, mean (95% CI)	529 (497–562)	< .001	502 (455–553)	559 (511–612)	.11
Age, years, median (IQR)	58.2 (52.6–62.8)	< .001	56.5 (52.2–62.2)	59.3 (55.7–63.1)	.23
HbA1c, mmol/mol, median (IQR)	36 (34–38)	-	36 (34–38)	36 (35–39)	.62
Smokers (current or previous),%	40.5%	-	41.7%	41.4%	-
Smoking pack years if smoker, median (IQR)	11.1 (7.5–20.0)	.27	9.8 (4.0–20.0)	13.5 (10.0–19.0)	.18

Lens fluorescence means and confidence intervals have been back-transformed from log-values. P-values from Student’s t-tests for normally distributed parameters (lens fluorescence, 120 min OGTT) and from Mann-Whitney-U tests for non-normally distributed parameters (age, HbA_1c_, smoking pack years). DZ: Dizygotic twins, IQR: Inter-quantile range, OGTT: Oral glucose tolerance test, MZ: Monozygotic twins.

**Fig 2:**
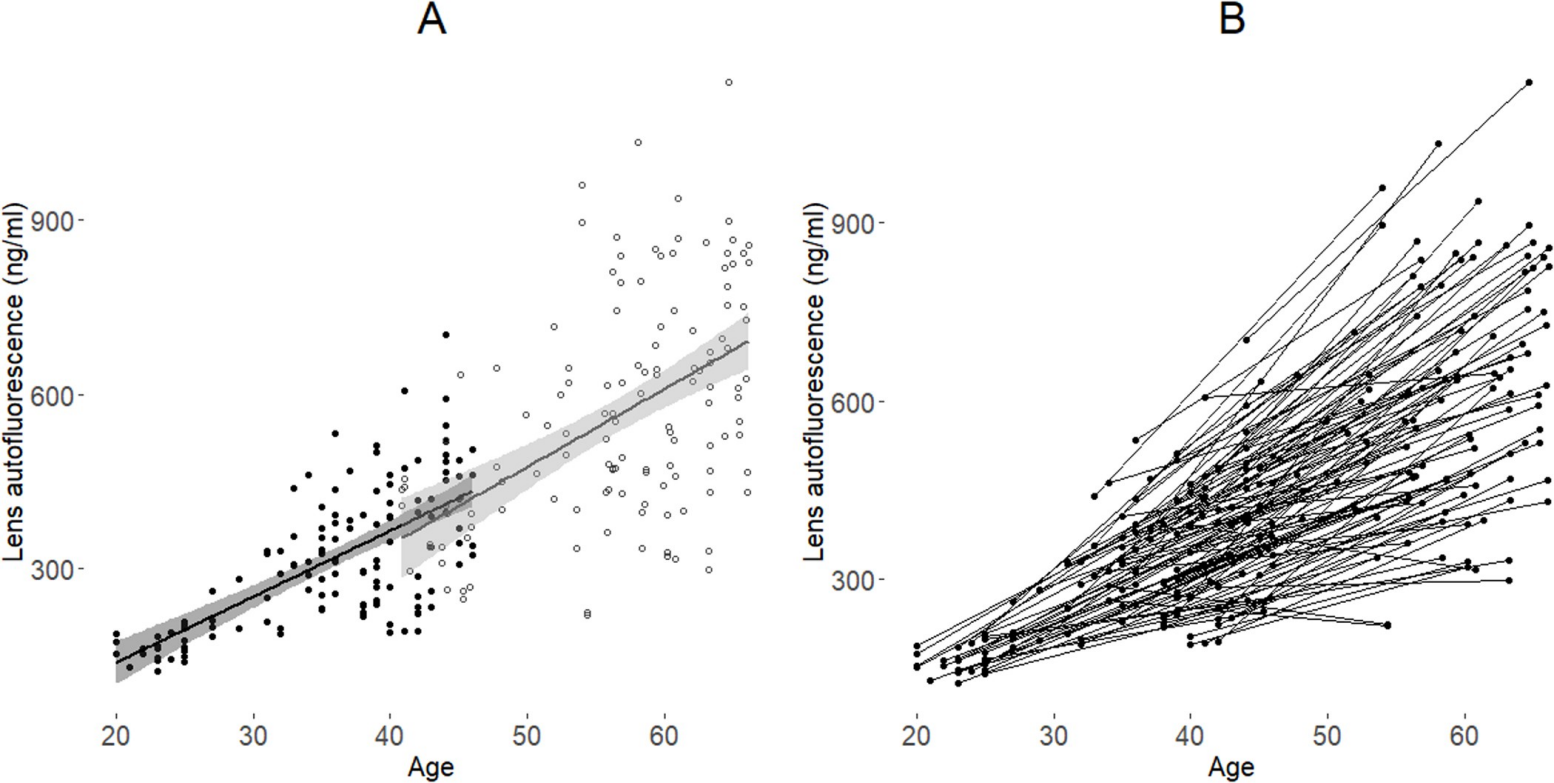
Lens fluorescence and age at the baseline and follow-up studies (n = 131). Linear regression lines with 95% confidence intervals for baseline data (A, black dots, black regression line) and follow-up data (A, hollow circles, grey regression line) and paired-measurements plot (B).

The interindividual variation in lens fluorescence increased with age, but there was an approximately linear continuation of increase in mean lens fluorescence with age between baseline and follow-up (Fig 2A, linear regression lines). Lens fluorescence had increased during the follow-up period in all but 3 (2.3%) subjects (Fig 2B).

Age and smoking pack years were significantly associated with lens fluorescence in both univariate and multivariable linear regression analyses at both baseline and at follow-up (p ≤ .041). In univariate analyses, age was the covariate that accounted for the biggest part of the variance in lens fluorescence (55.0% at baseline and 27.5% at follow-up, based on R^2^-values). Glycemia had no statistically significant effect on lens fluorescence in univariate models (p ≥ .069) and was omitted from multivariable analyses, which found that age and smoking pack years together accounted for 57.2% of the variance in lens fluorescence at baseline and 31.6% at follow-up (based on R^2^-values) (Table 2). Explorative analyses including both age, smoking pack years and glycaemia in multivariable regression analyses found that all three variables combined accounted for 58.3% of the variance in fluorescence at baseline and 32.2% at follow-up.

**Table 2:** Linear regression analyses of lens fluorescence (log10) as a function of covariates (n = 131).

Parameter \ analysis type	Univariate analyses			Multivariable analyses		
	Estimate (%*)	p-value	R^2^ (%)	Estimate (%*)	p-value	R^2^ (%)
**Baseline study (1998)**						
Age, ng/ml/year	1.7	< .001	55.0	1.7	< .001	57.2
Smoking, ng/ml/pack year	0.5	.002	2.5	0.5	.003	-
OGTT, ng/ml/mmol/L	1.3	.109	0.7	-	-	-
**Follow-up study (2019)**						
Age, ng/ml/year	1.1	< .001	27.5	1.1	< .001	31.6
Smoking, ng/ml/pack year	0.4	< .001	5.6	0.3	< .001	-
HbA_1c_, ng/ml/mmol/mol	0.7	.069	1.9	-	-	-

Results of log-level linear regression mixed model analyses adjusted for twin pair data clustering. Adjusted R^2^ values shown. *: Estimate coefficients have been exponentiated and converted to percentages, thereby indicating the percentage increase in median lens fluorescence when a given covariate (age, HbA_1c_, OGTT or smoking pack years) are increased by one unit (years, mmol/mol, mmol/L or pack years). **OGTT**: Oral glucose tolerance test glycemia (mmol/L) 2 hours after exposure.

In heritability analyses, the best fitting heritability models were the AE models at both baseline and follow-up, with ACE and ADE models being statistically non-inferior (for AIC values see Table 3). CE and DE models were statistically inferior models at both baseline (AIC -219.10 and -224.96 respectively) and follow-up (AIC -184.26 and -202.09 respectively).

**Table 3:** Broad-sense heritability analysis of lens fluorescence adjusted for age and smoking (n = 118).

Model \ coefficient	MZ correlation	DZ correlation	A (%)	D (%)	C (%)	E (%)	AIC
Baseline study (1998)							
ACE	0.91 [0.83–0.95]	0.62 [0.38–0.78]	57.2 [18.1–96.4]	-	33.5 [-5.7*-72.7]	9.3 [3.7–14.8]	-230.85
ADE	0.91 [0.83–0.95]	0.45 [0.43–0.48]	90.6 [85.2–95.9]	0.0 [0.0–0.0]	-	9.4 [4.1–14.8]	-228.90
AE	0.91 [0.83–0.95]	0.45 [0.43–0.48]	90.6 [85.2–95.9]	-	-	9.4 [4.1–14.8]	-230.90
**Follow-up study (2019)**							
ACE	0.93 [0.88–0.96]	0.57 [0.28–0.77]	72.3 [23.9–120.6*]	-	21.2 [-27.5*-69.8]	6.6 [2.7–10.5]	-204.32
ADE	0.93 [0.88–0.96]	0.47 [0.45–0.49]	93.3 [89.4–97.2]	0.0 [0.0–0.0]	-	6.7 [2.8–10.6]	-203.74
AE	0.93 [0.88–0.96]	0.47 [0.45–0.49]	93.3 [89.4–97.2]	-	-	6.7 [2.8–10.6]	-205.74

Brackets contain 95% confidence intervals. AE models were the best fitting models at both baseline and follow-up according to AIC.

*: Values of <0% or >100% are not practically meaningful but may arise as a technical consequence of the statistical analysis. **A:** Additive genetics (a^2^), **AIC:** Akaike information criterion, **ACE/ADE/AE**: Combinatory heritability models including either A and/or C and/or E. **C:** Shared environment (c^2^), **D:** Dominant genetics (D^2^), **DZ:** Dizygotic, **E:** Non-shared environment (e^2^), **MZ:** Monozygotic.

Dominant genetics (D) explained 0% of the variation in ADE models, which made heritability outcomes of ADE models equal to that of AE for each study visit. In the AE and ADE models, broad-sense heritability increased from 90.6% [95% CI 85.2–95.9] at baseline to 93.3% [CI 89.4–97.2] at follow-up at the expense of variance attributable to non-shared environmental factors, which showed a corresponding decrease from 9.4% [CI 4.1–14.8] to 6.7% [CI 2.8–10.6]. The ACE models showed a more pronounced increase in variance attributable to genetic factors from 57.2% [CI 18.1–96.4] at baseline to 72.3% [CI 23.9–120.6] follow-up. There was a trend toward lens fluorescence becoming more heritable between baseline and follow-up across all models, but no changes in heritability coefficients were statistically significant (Table 3).

## Discussion

This prospective study of the development of lens fluorescence over 21 years in 131 non-diabetic twins confirmed effects of age and smoking found in cross-sectional studies and showed that lens fluorescence remained more than 90% heritable. In the present analysis, where participants with diabetes were excluded, there was no detectable effect of current normal-range glycemia on lens fluorescence. A reduction by almost one half on the impact of age and cumulative smoking on lens fluorescence variance indicates that other influencing factors must have gained importance during the follow-up period. The persistently high heritability of lens fluorescence suggests that the effect may stem from genetic factors becoming more influential with age, including, perhaps, genes that dispose to or protect against cataract. Conversely, contributions to fluorescence from environmental factors unaccounted for in this study, such as cumulative photooxidation of lens crystallins by ultraviolet light, may be marginal. This is in line with findings in previous study, in which no increase in lens fluorescence was found in subjects with higher assumed life-time exposure to ultraviolet light [30].

Lens fluorescence increased by a mean of 11.8 ng/ml pr. year [95% CI 10.7–12.9] which is in reasonable agreement with a prior 13-year follow-up study of only 15 healthy participants, where the rate was mean 8.7 ng/ml pr. year [CI 7.3–10.1], given the difference in sample sizes (n = 131 vs n = 15) and minor methodological discrepancies (excitation and detection wavelengths 430–490 nm/530-630 nm versus 415–490 nm/510–550 nm) [31]. As expected, lens fluorescence increased from baseline to follow-up in the overall analysis [32], but there were 3 individuals (including one monozygotic twin pair) in whom it decreased. No abnormality of the lens was noted at slit-lamp biomicroscopy in these individuals and it remains for later follow-up to determine if this was the consequence of pre-cataractous lens opacification. Interindividual variation in lens fluorescence had increased with age, as seen in cross-sectional studies [33–36].

High lens fluorescence is associated with nuclear cataract [16,19–23], but high lens fluorescence and yellowing of the lens do not appear to be associated with visual complaints. As is the case with Scheimpflug densitometry, lens fluorometry may be superseded in clinical relevance by ray-tracing aberrometry, which is more directly able to quantify aberrations that affect the quality of image formation on the retina [37–39]

The relationship between lens fluorescence and accommodative amplitude has not been widely studied: Luo et al. examined healthy subjects, presumably in self-reported good health, as no inclusion or exclusion criteria were described and no assessment of glycemia was made [18]. They found no effect of lens fluorescence on accommodative amplitude. Adnan et al. studied young adults with and without type 1 diabetes and found a pronounced accommodative amplitude deficit in diabetes [40]. Together, the two studies indicate that either accommodative amplitude is independent of lens fluorescence, or a study of a population with a large age-independent variation in lens fluorescence, which could be realized by including subjects with diabetes, is needed to evaluate the full effect of lens fluorescence on accommodation. Obviously, fluorescence, browning and stiffening of the lens, although they all increase with age, may be mechanistically separate processes that do not respond in unison to changes in the environment of the lens or to external manipulation of the lens [41].

Although there is a continuing interest in the potential of lens fluorescence as a non-invasive diabetes screening modality or indirect marker of diabetic complications [35,42–45], it seems that lens fluorescence is too unreliable a marker to be of practical use. The explanation appears to be a ‘black box’ of genes that has a powerful influence on the rate at which fluorophores accumulate in the lens, of which we have found heritability (up to 93%) to be higher than both that of HbA_1c_ levels (75% [46]) and type 2 diabetes (up to 80% [47]). It remains to be determined if these genes have any influence on ocular or systemic health.

## Conclusion

From mid to late adulthood, over a period of 21 years, the level of blue-green fluorescence in the lens remained overwhelmingly heritable, but became markedly less predictable from age. Presumably, as the lens ages, its intrinsic characteristics come to dominate over environmental and systemic factors, perhaps in a prelude to the development of genetically determined cataract.

## Strengths and limitations

The present study is, to the best of our knowledge, the longest follow-up of lens fluorescence to date and its twin design has enabled statistically high-powered assessment of empirical heritability of a trait for which no obvious candidate genes are known.

Key risk factors have only been assessed at two study visits, one in self-reported terms (accumulative smoking), which may have been subject to recollection and interviewer biases.

Lens fluorescence was measured at baseline and follow-up using the same brand and model instrument [14]. A minor methodological difference was that no pharmacological pupil dilation was used at baseline, whereas it was used at follow-up, which is expected to have no meaningful influence on fluorescence readings, which are measured along the optical axis of the lens.

## References

[1] Charng J, Tan R, Luu CD, Sadigh S, Stambolian D, Guymer RH, et al. Imaging Lenticular Autofluorescence in Older Subjects. Invest Ophthalmol Vis Sci. 2017 Oct;58(12):4940–7. doi: 10.1167/iovs.17-22540 28973367 PMC5627676

[2] Singh R, Barden A, Mori T, Beilin L. Advanced glycation end-products: a review. Diabetologia [Internet]. 2001;44(2):129–46. Available from: http://10.0.3.239/s001250051591. doi: 10.1007/s001250051591 11270668

[3] Bron AJ, Vrensen GFJM, Koretz J, Maraini G, Harding JJ. The ageing lens. Ophthalmologica. 2000. 10.1159/000027475 10657747

[4] Fuentealba D, Friguet B, Silva E. Advanced glycation endproducts induce photocrosslinking and oxidation of bovine lens proteins through type-I mechanism. Photochem Photobiol. 2009. doi: 10.1111/j.1751-1097.2008.00415.x 18673320

[5] Monnier VM, Nagaraj RH, Portero-Otin M, Glomb M, Elgawish AH, Sell DR, et al. Structure of advanced Maillard reaction products and their pathological role. Nephrol Dial Transplant. 1996;11 Suppl 5:20–6. doi: 10.1093/ndt/11.supp5.20 9044302

[6] van Waateringe RP, Slagter SN, van der Klauw MM, van Vliet-Ostaptchouk J V, Graaff R, Paterson AD, et al. Lifestyle and clinical determinants of skin autofluorescence in a population-based cohort study. Eur J Clin Invest. 2016/03/24. 2016;46(5):481–90. doi: 10.1111/eci.12627 27002914 PMC5111733

[7] Uribarri J, Woodruff S, Goodman S, Cai W, Chen X, Pyzik R, et al. Advanced glycation end products in foods and a practical guide to their reduction in the diet. J Am Diet Assoc. 2010 Jun;110(6):911–16.e12. doi: 10.1016/j.jada.2010.03.018 20497781 PMC3704564

[8] Argirov OK, Lin B, Ortwerth BJ. Phototransformations of advanced glycation end products in the human eye lens due to ultraviolet A light irradiation. Ann N Y Acad Sci. 2005 Jun;1043:166–73. doi: 10.1196/annals.1333.021 16037236 PMC1564128

[9] Masaki H, Okano Y, Sakurai H. Generation of active oxygen species from advanced glycation end-products (AGEs) during ultraviolet light A (UVA) irradiation and a possible mechanism for cell damaging. Biochim Biophys Acta. 1999 Jun;1428(1):45–56. doi: 10.1016/s0304-4165(99)00056-2 10366759

[10] Kessel L, Kalinin S, Nagaraj RH, Larsen M, Johansson LB. Time-resolved and steady-state fluorescence spectroscopic studies of the human lens with comparison to argpyrimidine, pentosidine and 3-OH-kynurenine. Photochem Photobiol. 2002/12/05. 2002;76(5):549–54. doi: 10.1562/0031-8655(2002)076&lt;0549:trassf&gt;2.0.co;2 12462652

[11] Das BK, Sun TX, Akhtar NJ, Chylack LT, Liang JJN. Fluorescence and immunochemical studies of advanced glycation-related lens pigments. Investig Ophthalmol Vis Sci. 1998. 9761284

[12] Ranjan M, Beedu SR. Spectroscopic and biochemical correlations during the course of human lens aging. BMC Ophthalmol. 2006. doi: 10.1186/1471-2415-6-10 16519820 PMC1450316

[13] Lynnerup N, Kjeldsen H, Heegaard S, Jacobsen C, Heinemeier J. Radiocarbon dating of the human eye lens crystallines reveal proteins without carbon turnover throughout life. PLoS One. 2008/01/31. 2008;3(1):e1529. doi: 10.1371/journal.pone.0001529 18231610 PMC2211393

[14] Kessel L, Hougaard JL, Sander B, Kyvik KO, Sorensen TI, Larsen M. Lens ageing as an indicator of tissue damage associated with smoking and non-enzymatic glycation—a twin study. Diabetologia. 2002/10/16. 2002;45(10):1457–62. doi: 10.1007/s00125-002-0925-3 12378389

[15] Calvo-Maroto AM, Perez-Cambrodi RJ, Garcia-Lazaro S, Ferrer-Blasco T, Cervino A. Ocular autofluorescence in diabetes mellitus. A review. J Diabetes. 2016/05/06. 2016;8(5):619–28. doi: 10.1111/1753-0407.12423 27147470

[16] Suarez G, Oronsky AL, Koch MHLJ. Age-dependent structural changes in intact human lenses detected by synchrotron radiation x-ray scattering. Correlation with Maillard reaction protein fluorescence. J Biol Chem. 1993. 8349657

[17] Hemenger RP, Occhipinti JR. Is accommodative amplitude correlated with lens fluorescence? Optom Vis Sci. 1990. doi: 10.1097/00006324-199011000-00014 2250897

[18] Luo X, Kymes SM, Gordon MO, Bassnett S. Lens fluorescence and accommodative amplitude in pre-presbyopic and presbyopic subjects. Exp Eye Res. 2007. doi: 10.1016/j.exer.2007.01.012 17359974 PMC2682368

[19] Siik S, Chylack LT, Friend J, Wolfe J, Teikari J, Nieminen H, et al. Lens autofluorescence and light scatter in relation to the lens opacities classification system, LOCS III. Acta Ophthalmol Scand. 1999. 10.1034/j.1600-0420.1999.770504.x 10551289

[20] Gakamsky DM, Dhillon B, Babraj J, Shelton M, Smith SD. Exploring the possibility of early cataract diagnostics based on tryptophan fluorescence. J R Soc Interface. 2011. doi: 10.1098/rsif.2010.0608 21508010 PMC3177609

[21] Erichsen JH, Mensah A, Kessel L. Non-invasive tryptophan fluorescence measurements as a novel method of grading cataract. Exp Eye Res. 2017. doi: 10.1016/j.exer.2017.09.006 28935513

[22] Gakamsky A, Duncan RR, Howarth NM, Dhillon B, Buttenschön KK, Daly DJ, et al. Tryptophan and Non-Tryptophan Fluorescence of the Eye Lens Proteins Provides Diagnostics of Cataract at the Molecular Level. Sci Rep. 2017. doi: 10.1038/srep40375 28071717 PMC5223181

[23] Siik S, Airaksinen PJ, Tuulonen A, Nieminen H. Autoflourescence in cataractous human lens and its relationship to light scatter. Acta Ophthalmol. 1993. 10.1111/j.1755-3768.1993.tb07153.x 8362640

[24] Rovati L, Docchio F. Autofluorescence methods in ophthalmology. J Biomed Opt. 2004/01/13. 2004;9(1):9–21. doi: 10.1117/1.1628241 14715054

[25] Sepah YJ, Akhtar A, Sadiq MA, Hafeez Y, Nasir H, Perez B, et al. Fundus autofluorescence imaging: Fundamentals and clinical relevance. Saudi J Ophthalmol. 2014/05/21. 2014;28(2):111–6. doi: 10.1016/j.sjopt.2014.03.008 24843303 PMC4023118

[26] Kolomeyer AM, Nayak N V, Szirth BC, Khouri AS. Fundus autofluorescence imaging in an ocular screening program. Int J Telemed Appl. 2013/01/15. 2012;2012:806464. doi: 10.1155/2012/806464 23316224 PMC3536047

[27] Brauer JL, Schultz R, Klemm M, Hammer M. Influence of lens fluorescence on fluorescence lifetime imaging ophthalmoscopy (Flio) fundus imaging and strategies for its compensation. Transl Vis Sci Technol. 2020. doi: 10.1167/tvst.9.8.13 32855860 PMC7422756

[28] Skytthe A, Christiansen L, Kyvik KO, Bodker FL, Hvidberg L, Petersen I, et al. The Danish Twin Registry: Linking surveys, national registers, and biological information. Twin Res Hum Genet. 2013. 10.1017/thg.2012.77 PMC3629375 23084092

[29] Zeimer RC, Noth JM. A new method of measuring in vivo the lens transmittance, and study of lens scatter, fluorescence and transmittance. Ophthalmic Res. 1984/01/01. 1984;16(5):246–55. doi: 10.1159/000265325 6483379

[30] Kessel L, Kofoed PK, Zubieta-Calleja G, Larsen M. Lens autofluorescence is not increased at high altitude. Acta Ophthalmol. 2009/05/07. 2010;88(2):235–40. doi: 10.1111/j.1755-3768.2008.01488.x 19416110

[31] Van Best JA, Van Delft JL, Keunen JE. Long term follow-up of lenticular autofluorescence and transmittance in healthy volunteers. Exp Eye Res [Internet]. 1998 Jan;66(1):117–23. Available from: https://www.ncbi.nlm.nih.gov/pubmed/9533837. doi: 10.1006/exer.1997.0417 9533837

[32] Lynnerup N, Kjeldsen H, Heegaard S, Jacobsen C, Heinemeier J. Radiocarbon dating of the human eye lens crystallines reveal proteins without carbon turnover throughout life. PLoS One. 2008. doi: 10.1371/journal.pone.0001529 18231610 PMC2211393

[33] Koefoed Theil P, Kessel L, Hansen T, Lund-Andersen H, Pedersen O, Larsen M. Lens fluorescence in relation to glucose tolerance and genetic predisposition to type 2 diabetes mellitus in a population-based study. Curr Eye Res. 2006/09/13. 2006;31(9):733–8. doi: 10.1080/02713680600850971 16966146

[34] Burd J, Lum S, Cahn F, Ignotz K. Simultaneous noninvasive clinical measurement of lens autofluorescence and rayleigh scattering using a fluorescence biomicroscope. J Diabetes Sci Technol. 2013/01/09. 2012;6(6):1251–9. doi: 10.1177/193229681200600603 23294769 PMC3570864

[35] Cahn F, Burd J, Ignotz K, Mishra S. Measurement of Lens Autofluorescence Can Distinguish Subjects With Diabetes From Those Without. J Diabetes Sci Technol. 2014/05/31. 2014;8(1):43–9. doi: 10.1177/1932296813516955 24876536 PMC4454118

[36] Siik S, Airaksinen PJ, Tuulonen A, Alanko HI, Nieminen H. Lens autofluorescence in healthy individuals. Acta Ophthalmol. 1991. doi: 10.1111/j.1755-3768.1991.tb02709.x 1872137

[37] Faria-Correia F, Ramos I, Lopes B, Monteiro T, Franqueira N, Ambrosio R Jr. Correlations of Objective Metrics for Quantifying Dysfunctional Lens Syndrome With Visual Acuity and Phacodynamics. J Refract Surg. 2017/02/14. 2017;33(2):79–83. doi: 10.3928/1081597X-20161206-05 28192585

[38] Faria-Correia F, Ramos I, Lopes B, Monteiro T, Franqueira N, Ambrosio R Jr. Comparison of Dysfunctional Lens Index and Scheimpflug Lens Densitometry in the Evaluation of Age-Related Nuclear Cataracts. J Refract Surg. 2016/04/14. 2016;32(4):244–8. doi: 10.3928/1081597X-20160209-01 27070231

[39] Li Z, Yu L, Chen D, Chang P, Wang D, Zhao Y, et al. Dysfunctional Lens Index Serves as a Novel Surgery Decision-Maker for Age-Related Nuclear Cataracts. Curr Eye Res. 2019. doi: 10.1080/02713683.2019.1584676 30822168

[40] Adnan Efron N, Mathur A, Edwards K, Pritchard N, Suheimat M, et al. Amplitude of accommodation in type 1 diabetes. Invest Ophthalmol Vis Sci. 2014/10/10. 2014;55(10):7014–8. doi: 10.1167/iovs.14-15376 25298413

[41] Kessel L, Eskildsen L Fau—van der Poel M, van der Poel M Fau—Larsen M, Larsen M, One PL. Non-invasive bleaching of the human lens by femtosecond laser photolysis. 2010;(1932–6203 (Electronic)). doi: 10.1371/journal.pone.0009711 20300521 PMC2838787

[42] Sertbas M, Sertbas Y, Uner OE, Elarslan S, Okuroglu N, Ak F, et al. Lens autofluorescence ratio as a noninvasive marker of peripheral diabetic neuropathy. Polish Arch Intern Med. 2019. doi: 10.20452/pamw.4449 30762026

[43] Pehlivanoglu S, Acar N, Albayrak S, Karakaya M, Ofluoglu A. The assessment of autofluorescence of the crystalline lens in diabetic patients and healthy controls: can it be used as a screening test? Clin Ophthalmol. 2018/07/10. 2018;12:1163–70. doi: 10.2147/OPTH.S164960 29983542 PMC6027705

[44] Karumanchi DK, Gaillard ER, Dillon J. Early Diagnosis of Diabetes through the Eye. Photochem Photobiol. 2015 Nov;91(6):1497–504. doi: 10.1111/php.12524 26313889

[45] Stirban A. Measurement of Lens Autofluorescence for Diabetes Screening. J Diabetes Sci Technol. 2014/05/31. 2014;8(1):50–3. doi: 10.1177/1932296813514501 24876537 PMC4454121

[46] Simonis-Bik AMC, Eekhoff EMW, Diamant M, Boomsma DI, Heine RJ, Dekker JM, et al. The heritability of HbA1c and fasting blood glucose in different measurement settings. Twin Res Hum Genet. 2008. doi: 10.1375/twin.11.6.597 19016616

[47] Ali O. Genetics of type 2 diabetes. World J Diabetes [Internet]. 2013 Aug 15;4(4):114–23. Available from: https://pubmed.ncbi.nlm.nih.gov/23961321. doi: 10.4239/wjd.v4.i4.114 23961321 PMC3746083

